# A novel approach of using transtibial transport (TTT) to manage thromboembolic events following surgical management of necrotizing soft tissue infection: a case report

**DOI:** 10.3389/fmed.2024.1481388

**Published:** 2025-01-07

**Authors:** Johnson Boey, Jordon Lee, ZheGang Zhou

**Affiliations:** ^1^Dr Foot Podiatry Clinic, Singapore, Singapore; ^2^Department of Hand and Microsurgery, Peking University Shenzhen Hospital, Shenzhen, China

**Keywords:** diabetic limb salvage, pulmonary embolism, acute limb ischemia, gangrene, transtibial transport, bone transport

## Abstract

Necrotising soft tissue infections (NSTIs) are one of the most challenging and severe forms of infections. The prognosis requires accurate and aggressive diagnosis and management. In this case, we present an unexplained case of concurrence of TE events following BKA for the surgical management of NSTI. As with the standard management, the first step involves aggressive surgical debridement of non-viable tissue which eventually lead to below-knee amputation for effective source control. Lower limb endovascular angioplasty was attempted but unsuccessful. The PAD was managed with antithrombotic therapy. In spite of this, the patient developed thromboembolic events 1 week following BKA. In response, the antiplatelet therapy with low-molecular weight heparin was enhanced. Transtibial transport was performed after patient found to have stenosis in distal tibial arteries, which the patient refused any further vascular intervention. Nonetheless, with meticulous planning and concerted team efforts, we successfully reversed TE events and salvaged the contralateral limb without the need for amputation. With intensive rehabilitation, the patient was able to return to their pre-morbid functional quality of life.

## 1 Introduction

Necrotising soft tissue infection (NSTI), originally described as gas gangrene, is a severe soft tissue infection affecting approximately three per 100,000 individuals globally (1, 2) whereas significant higher prevalence has been reported in Thailand (3). The prognosis of NSTI is generally complicated by morbidity due to amputation, multi-organ dysfunction associated with septic shock, and mortality in one-third of cases (4). The key to managing NSTI is early diagnosis and aggressive intervention, as delays in intervention can lead to morbidity and mortality (5). The close resemblance of its clinical symptoms with soft tissue infections (SSI) makes timely diagnosis challenging. Medical imaging can beneficial but their findings are limited to advanced stages of NSTI (6). The Laboratory Risk Indicator for Necrotising Fasciitis (LRINEC) score derived from six common laboratory biomarkers have been used to stratify the probability of NSTI to aid in diagnosis of NSTI (7). The appropriate treatment of NSTI necessitate aggressive surgical exploration and systemic antibiotic regimens are required for effective source control.

The rapid spread of tissue necrosis has been linked to infection-induced thrombosis through the activation of platelets and neutrophil extracellular traps (NETs) (8, 9). However, the concurrence of cutaneous necrosis with thromboembolic (TE) events such as pulmonary embolism (PE) and acute limb ischemia (ALI) has been uncommon. In this case report, we presented an unexplained case of PE and ALI, which occurred despite timely management of NSTI and medical management of peripheral arterial disease (PAD) after a failed angioplasty attempt.

## 2 Surgical technique of transtibial transport (TTT)

**FIGURE 1:**
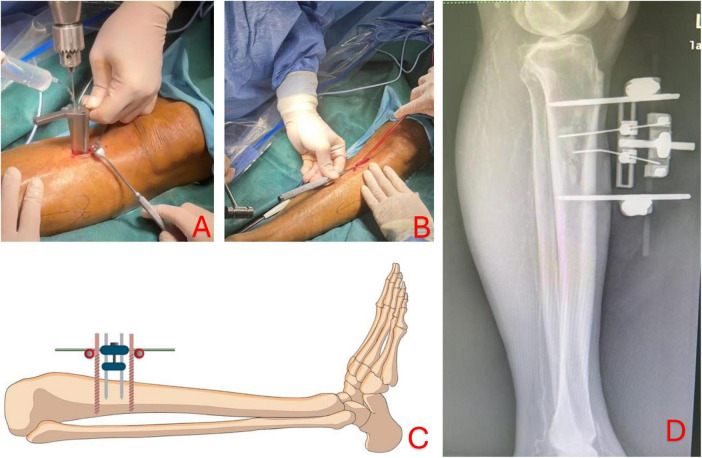
Corticectomy is performed by drilling multiple holes into the cortex to create a rectangular block of 5 cm by 3 cm **(A)**; osteotome is then used to lift the cortex carefully while avoiding causing fracture to the long bone structure **(B)**; two 2-mm pins (yellow) are inserted into the bone cortex for subsequent osseous distraction and two 4-mm pins (red) are drilled into bone shaft to offer greater stability of the construct **(C)**. Lateral view of lower limb showing TTT construct after medial and lateral distraction before removal of fixation **(D)**.

Transtibial transport (TTT) is a surgical technique evolved around the concept of distraction osteogenesis. The gradual mechanical displacement of cortex, as described below, stimulate osteogenic activity and tissue regeneration (10). The surgical protocol described in this paper is adapted from the recent clinical guideline written by the Chinese Association of Orthopaedic Surgeons (CAOS) (11). An incision approximately 5 cm in length was made, approximately 3cm from the tibial tuberosity, to expose the periosteum. A corticectomy block, approximately 5cm in length and 3cm in width, is created by drilling multiple holes into the cortex (Figure 1A) and osteotome is used to carefully lift the cortex while avoiding causing fracture to the long bone (Figure 1B). Two 2-mm steinmann pins spaced at 2-cm apart were drilled into cortex within the corticectomy block for distraction, and two 4-mm steinmann pins were inserted through the tibial shaft outside of the corticetomy block for stabilization of the construct (Figure 1C). The TTT fixation construct was assembled using CareFix system (Shanghai Medical Instrument Co., Ltd). Transverse distraction was commenced 5 days after the instillation, at rate of 1 mm per day in the lateral direction for the first 10 days and followed by the reverse (medial) direction in the next 10 day. Xrays in lateral view of lower limb was taken after 2 weeks after the completion of distraction prior to the removal of the construct (Figure 1D). The external fixation construct for TTT was removed 1 week after the completion of the distraction phase.

## 3 Case description

**FIGURE 2:**
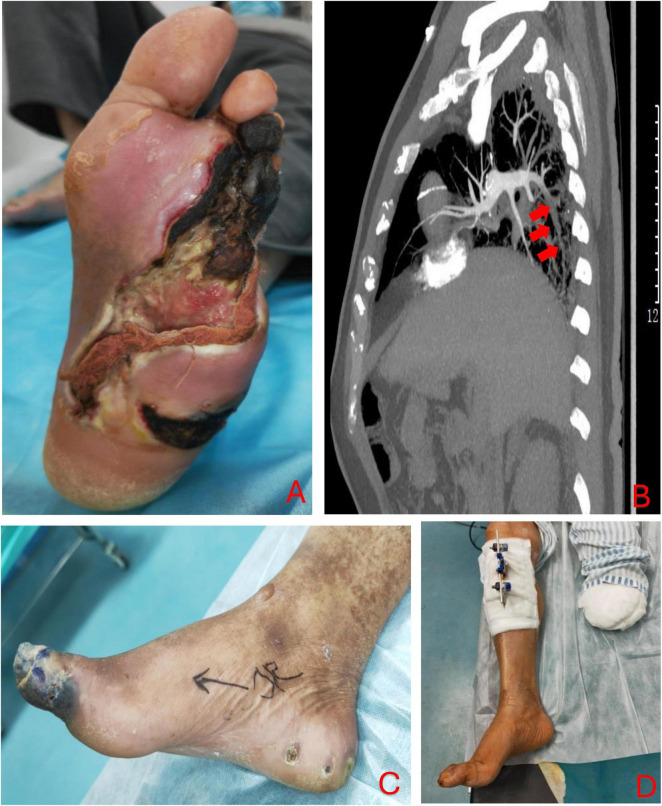
The initial presentation of left foot at the time of admission **(A)**; CTA scan revealed emboli in the posterior basal branch of pulmonary artery to right lower lobe (as marked by the red arrows) **(B)**; acute limb ischemia in right lower limb despite on anticoagulation therapy **(C)**. Transtibial transport (TTT) in the right lower limb was done and the tissue perfusion was restored which reversed the hallux necrosis **(D)**.

A 70-year-old Chinese man presented with chronic foot ulceration with multiple gangrenous toes (Figure 2A), on the background of poorly controlled type 2 diabetes mellitus (T2DM), peripheral arterial disease (PAD) and left 5th toe amputation. Patient has familial history of T2DM, coronary artery disease (CAD) and PAD. His current medication includes metformin and asprin for long-term management of T2DM and PAD. Physical examination revealed extensive tissue defect in the forefoot, purulent pus discharge from wound edges, and spreading lymphangitis in the absence of pedal pulses. Laboratory investigations revealed anemia, hyperglycemia (270 mg/dL), elevated levels of inflammatory markers, from which the LINREC score was determined as 7 (Table 1). Blood cultures were negative. Liver and renal panel is normal. Arterial duplex showed multiple stenoses in the posterior and anterior tibial arteries (ATA) of the left lower limb. The patient underwent immediate incisional and drainage (I&D), amputation of 3rd and 4th rays and eventually below-knee amputation (BKA) due to worsening infection and ischemia. Deep-tissue culture was taken which grew *methicillin-resistant Staphylococcus aureus(MRSA)*. IV vancomycin was administered for 2 weeks and later stepdown to oral clindamycin for another 4-weeks. An attempt of endovascular angioplasty was done but unable to re-canalize both the affected arteries. To optimize antithrombotic effect, low-molecular-weight heparin (LMWH) was added to current antiplatelet regimen. One week following BKA, the patient developed thromboembolic (TE) events; emboli was in the posterior basal segment of the pulmonary artery to the right lower lobe on computed tomography Angiography (CTA) (Figure 2B), and acute limb ischemia (ALI) in the right first toe (Figure 2C). The D-dimer level was found to be 1.02 mg/L. Upon confirmation of pulmonary embolism on computed tomographic angiography (CTA) and positive D-dimer result, the dosage of LMWH was doubled to 0.4 ml bd. TTT was performed in the right lower limb after recent discovery of stensoses in distal ATA and the patient declined further vascular interventions. The patient was informed of the risk involved with TTT over traditional endovascular or open vascular interventions. The pulmonary emboli (PE) were successfully removed and right first toe return to normal (Figure 2D). Vascular assessment of right first toe found toe pressure of 70 mmHg, capillary refill less than 3 s, and biphasic waveform on ultrasound doppler, all of which indicate sufficient arterial perfusion to the first toe. At the 24-month follow-up visit, the patient had fully recovered and was ambulating well with a prosthetic limb for left BKA. He was very satisfied with the outcome of TTT, in that, toe ischemia was resolved without amputation and further vascular interventions. He hope such technique could be offered to other patients with similar clinical presentations over lower limb amputations.

**TABLE 1:** The Laboratory Risk Indicator for Necrotising Fasciitis (LRINEC) system is based on six laboratory parameters: c-reactive protein (CRP), white blood cell (WBC) count, creatinine, hemoglobin, glucose and sodium.

Laboratory parameters	Value	Score
C-reactive protein (CRP)	350 mg/L	4
White blood cell (WBC) count	21 × 10^4^/mm^3^	1
Creatine	0.81 mg/dL	0
Hemoglobin	11.3 g/dL	1
Glucose	270 mg/dL	1
Sodium	136 mmol/L	0
Total	7 (moderate risk)	

This score stratified the risk of NSTI into three category; low risk (<5): 50% probability; moderate risk (≤7): 50–75% probability; high (>8): >75% probability. The LRINEC score for this patient is calculated to be 7 which carried moderate risk (<75% probability) of having NSTI.

## 4 Discussion

NSTIs represent a compendium of soft tissue infections marked by rapid necrosis involving the skin and subcutaneous tissue (necrotizing cellulitis), fascia (necrotizing fasciitis), and muscle (myonecrosis or gas gangrene) (12–14). NSTI are predominately polymicrobial (Type I) involving both aerobic and anaerobic pathogens, which has been associated with several comorbidities, such as diabetes mellitus (DM), obesity, chronic kidney disease, and immunocompromised status (12, 13). Occurring to a lesser extent, the monomicrobial are further divided into three distinct microbial profiles: Group A *Streptococcus*/*Staphylococcus aureus* (Type II), *Clostridium/Vibrio spp.* (Type III), and *candida spp.* (Type IV) (14). The natural history of NSTI shares a common inflammatory pathway with other non-infectious origins, such as trauma or lymphedema, resulting in misdiagnosis over 70% of the time (4). As the disease progresses, an exuberant inflammatory response leads to the rapid development of tissue necrosis, tenderness may become disproportionate (“crescendo pain”), and bullae and crepitus may occur (12, 13).

To date, no single diagnostic test has accurately identified NSTI beyond reasonable doubt. Laboratory findings of altered coagulation profiles, elevated levels of C-reactive protein (CRP), leukocytosis, and hyperglycemia are common; however, they do not sufficiently distinguish it from other SSIs (14). Wall et al. (15) found that white blood cell (WBC) (>15,000 cells/mm^3^) and sodium (>135 mmol/L) provide a sensitivity of 90% and a negative predictive value (NPV) of 99% in identifying NSTI. In addition, hypoalbuminemia and elevated creatinine levels may be observed in patients with existing kidney disease. Wong et al. (7) conceptualized a Laboratory Risk Indicator for Necrotising Fasciitis (LRINEC), a 13-point scoring system based on six laboratory parameters (Table 1), to simplify and improve the diagnosis of NSTI. A score ≥ of 6 produced a positive predictive value (PPV) and NPV of 92 and 96%, respectively (7). At this threshold, systematic reviews have reported a sensitivity of 43–80%, specificity of 84.8%, PPV of 57–64%, and NPV of 42–86% for diagnosing NSTI (16, 17). A higher LRINEC ≥ 8 may help rule out NSTI with a specificity of more than 90% (17). The appropriate management of NSTI involves aggressive debridement, antibiotic therapy, and resuscitation. Moreover, the severity and duration of the intervention greatly influenced the prognosis of NSTI. For instance, the mortality rate doubled in patients with an LRINEC score > 6, while surgical intervention within 6 h of initial presentation result in a 40% reduction in the mortality rate (18). In our case, there were extensive tissue necrosis, purulent pus discharge and LRINEC score of 7 at the time of admission. These findings led to prompt action by our team, involving both surgical interventions and systemic pharmacotherapy.

Despite our best effort in perioperative management, the patient developed thromboembolic events. PE is a common perioperative complication that often occurs within the first 6 weeks after surgery (19, 20). The annual incidence of PE is approximately 100 per 100,000 persons (20). Nonetheless, owing to its complexity, accurate diagnosis of PE is difficult, and the mortality rate is approximately 20% within 3 months, even with appropriate management (19). The treatment strategy relies on risk stratification, ranging from immediate thrombolytic therapy for high-risk patients to LMWH and/or oral anticoagulant therapy for intermediate- to low-risk patients. In our case, the patient developed TE events 1 week after BKA, while receiving antithrombotic therapy following an unsuccessful endovascular angioplasty. After assessing the risk of clinical deterioration and the absence of additional thrombi in the lower limb, TE event was managed with anticoagulation. The concurrent development of PE and ALI in the side may seem coincidental and unexpected initially, it was later believed to be related as both events occurred in the ipsilateral side and following recent major surgery such as BKA. However, there were no sufficient conclusive evidence to substantiate such proposition. The resolution of TE events rest upon on appropriate antithrombotic regimen, in this case, consist of aspirin and LMWH.

Due to the patient’s reluctance to undergo vascular intervention for the right lower limb, TTT was considered. Since its inception, TTT has been widely used in the treatment of ischemic foot disease and non-healing wounds (21). It leverages the potential of upregulating multiple pro-angiogenic growth factors to systematically improve microcirculation at the cellular level (21). Common risk associated with TTT include cortical bone fracture and fixation-associated infection (21). TTT procedure is avoided during the initial infected phase. In our case, the TTT procedure was done approximately 2-weeks after eradication of deep-tissue infection. Secondly, the TTT was recommended following the new stenosis in the ATA, to which the patient refused further vascular intervention. During the application of external fixation construct for TTT procedure, inflammatory biomarkers were normal and no evidence of re-infection was found. The clinical effect of TTT on the resolution of TE events remains unclear. In our case, prompt detection and early intervention of TE events using antithrombotic plays a key role in their resolution.

The catastrophic nature of NSTI cannot be overstated; it requires prompt diagnosis and aggressive intervention. Given appropriate management and rehabilitation, patients can often return to their pre-morbid quality of life. The occurrence of two TE events in the ipsilateral side is uncommon but possible, especially after major surgery such as BKA. The urgency of intervening TE event is paramount as delay in treatment will result in poor prognosis. The novelty in our management protocol is the dual approach of improving arterial circulation with TTT while optimizing antithrombotic therapy for the management of TE events. Moreover, the role of TTT on the prevention of diabetic limb complication is in its infancy and will requires a more robust investigation to establish its credibility.

## Data Availability

The datasets presented in this study can be found in online repositories. The names of the repository/repositories and accession number(s) can be found in this article/supplementary material.
